# Adequacy of in-mission training to treat tibial shaft fractures in mars analogue testing

**DOI:** 10.1038/s41598-023-43878-1

**Published:** 2023-10-23

**Authors:** Julie Manon, Michael Saint-Guillain, Vladimir Pletser, Daniel Miller Buckland, Laurence Vico, William Dobney, Sarah Baatout, Cyril Wain, Jean Jacobs, Audrey Comein, Sirga Drouet, Julien Meert, Ignacio Sanchez Casla, Cheyenne Chamart, Jean Vanderdonckt, Olivier Cartiaux, Olivier Cornu

**Affiliations:** 1 Université Catholique de Louvain (UCLouvain), Louvain-La-Neuve, Belgium; 2 UCLouvain - IREC, Morphology Lab (MORF), Avenue Emmanuel Mounier 52 - B1.52.04, 1200 Brussels, Belgium; 3 UCLouvain - IREC, Neuromusculoskeletal Lab (NMSK), Brussels, Belgium; 4 Orthopaedic Surgery Department, Cliniques Universitaires Saint-Luc, Brussels, Belgium; 5 Crew 227 – Mission Analogue Research Simulation (M.A.R.S. UCLouvain) – Mars Desert Research Station (MDRS), Utah, USA; 6 European Space Agency (Ret.), Blue Abyss, UK; 7 Human System Risk Board (HSRB), NASA Johnson Space Center, Houston, TX USA; 8 Department of Emergency Medicine, Duke University, North Carolina, USA; 9 INSERM, Mines Saint-Étienne, Univ Jean Monnet, U 1059 Sainbiose, 42023 Saint-Étienne, France; 10 Radiobiology Unit, Belgian Nuclear Research Centre, SCK CEN, Mol, Belgium; 11 School of Aeronautical, Automotive, Chemical and Materials Engineering, Loughborough University, Loughborough, UK; 12 Department of Health Engineering, ECAM Brussels Engineering School, Haute Ecole “ICHEC-ECAM-ISFSC”, Brussels, Belgium

**Keywords:** Fracture repair, Orthopaedics, Astronomy and planetary science

## Abstract

Long bone fractures are a concern in long-duration exploration missions (LDEM) where crew autonomy will exceed the current Low Earth Orbit paradigm. Current crew selection assumptions require extensive complete training and competency testing prior to flight for off-nominal situations. Analogue astronauts (n = 6) can be quickly trained to address a single fracture pattern and then competently perform the repair procedure. An easy-to-use external fixation (EZExFix) was employed to repair artificial tibial shaft fractures during an inhabited mission at the Mars Desert Research Station (Utah, USA). Bone repair safety zones were respected (23/24), participants achieved 79.2% repair success, and median completion time was 50.04 min. Just-in-time training in-mission was sufficient to become autonomous without pre-mission medical/surgical/mechanical education, regardless of learning conditions (p > 0.05). Similar techniques could be used in LDEM to increase astronauts’ autonomy in traumatic injury treatment and lower skill competency requirements used in crew selection.

## Introduction

Future crewed missions beyond low earth orbit will compel researchers and scientists to question our understanding of the health, safety and autonomy of astronauts and to devise new strategies to guarantee their safety and survival chances^1,2^. Nowadays, crew selection for future missions relies on the Knowledge – Skill – Ability – Other traits (KSAOs) concept^3^. However, maximizing the effectiveness and cost-efficiency of astronaut education requires considering the time and ease of acquiring valuable skills. This study focuses on this new and additional insight applied on long bone fracture repair capability, which is merely an example to illustrate the overall approach to any capability.

The decrease in bone mineral density (BMD)^4–9,11^ is a major health concern for astronauts ^4–9^ encountered during long-duration exploration missions (LDEM) (defined by NASA as longer than 30 days^10^), occurring as early as the first month of microgravity ^4,7,8^. Astronauts’ BMD continues to decline by 1 to 2.2% per month ^9,10^, similar to the annual decrease in postmenopausal women^11^, rendering their bones more fragile and prone to fracturing upon return to gravity^1,9,10,12–14^. After a long duration interplanetary journey in weightlessness, landing on Mars, with a gravity field a third of that on Earth, would increase the risk of fractures when the astronauts with reduced BMD carry out demanding tasks on the surface of the red planet^9^. A Bone Fracture Risk Model (BFxRM) was developed by NASA to compute the fracture risk based on patient characteristics (e.g. age, sex, initial BMD), duration of space missions and cumulative microgravity exposures, as well as shocks and protective load absorption of specific activities and/or equipment^15^. This study showed that the risk of fractures on Mars was higher due to weakened bone integrity, with the wrist in the first position^15^. Lower extremity stress fracture are also much more frequent than hip/proximal femur fracture in partial gravity environments^16^. Physical exercise and pharmacological prevention, advised in order to limit the BMD loss, may not counteract it completely^4,6,12,13^. Despite their exercise programs, 92% of astronauts on board of Mir and/or ISS space stations suffered a minimum of 5% BMD loss in at least one skeletal area^12^, and 40% experienced 10% or more BMD loss throughout the body^12^.

The occurrence of a long bone fracture in space, coupled with the lack of medical orthopaedic expertise, could endanger the health and/or life of the injured astronaut and compromise the whole mission. An untreated fracture presents risks of haemorrhage, infection, fat embolism and fat embolism syndrome, all of which can sometimes be life-threatening, and later, risks of non-consolidation, malunion and, ultimately, major loss of autonomy^17–19^.

Given a journey of six to eight months to return from Mars to Earth, an expedited abort return is impossible^20^. Although telesurgery has improved, transmission delays (e.g. 6 to 44 min between Earth and Mars, depending on relative planetary positions) remains problematic for performing remote surgery by an Earth surgeon or even for tele-mentoring (surgery performed by astronaut with the guidance of an Earth surgeon)^1,2,9,20,21^. Remote astronaut crews will require medical autonomy to diagnose and treat injuries^20^.

To our knowledge, little is known about bone remodelling and fracture repair in a space environment, the optimal treatment for healing long bone fractures on Mars (cast, internal plating, intramedullary nailing…) and the impact of surgery in weightlessness^9,22^. BMD loss is known to persist after returning to Earth, and the reversal of these changes can be slow suggesting that the time needed for complete bone regeneration can be longer than the mission itself^5,8^. The consolidation rate of a bone fracture seems to be lower in microgravity than on Earth with a smaller and weaker callus^1,23^. Achieving sufficient stability through orthopaedic treatment is crucial for promoting fracture consolidation along the correct axis, thereby avoiding potential complications like non-union or infection. This can be possible with all classical orthopaedic procedures ^24,25^, nevertheless, space is more demanding. Amongst others, the external fixator stands as the treatment of choice for space applications, capable of managing all types of fractures in a hostile environment characterized by limited resources and the absence of healthcare facilities to address complications.

Regarding generalised trauma, tibial shaft fractures are among the commonest open or closed long bones fractures on Earth^24,26,27^ and are conventionally treated using various orthopaedic surgical procedures, including the external fixator. Our study model focuses on the lower limb. A metallic device, consisting of pins inserted into the bone, connected to rods outside the leg, offers strength and support for the healing bone and surroundings. We propose the application of an easy-to-use external fixator (EZExFix)^28,29^ procedure under realistic operational conditions on Mars, and have assessed it during a two weeks simulation mission at the Mars Desert Research Station (MDRS, Utah). The goal was to offer astronauts the opportunity to learn, during the mission, in an accelerated manner the procedure to set up the EZExFix, without relying on Earth-based support and without requiring extensive prior surgical training. We then evaluated effectiveness and autonomy of each astronaut during the surgery, as well as safety of the injured astronaut. Broadening the scope of the study, the need for a medical and/or mechanical/engineering background, and the reaction to the stressful conditions imposed by the mission were evaluated. This approach could improve the cost-effectiveness of meeting KSAOs requirements.

## Material and methods

### Study design

Six analogue astronauts from crew 227 of the Tharsis mission (2022) at the MDRS gave written informed consent to participate in this research into bone fracture surgery. The methods were performed in accordance with relevant guidelines and regulations and approved by the hospital-faculty ethics committee of the Cliniques Universitaires Saint-Luc, Belgium (N°B403201523492).

#### Knowledge group

Astronauts were divided into 3 groups according to their educational background. The first group called “Anat” was somewhat skilled in human anatomy (studies in the medical or biomedical field but not in surgery). The second group called “Meca”, had knowledge of mechanics, stability, forces movements and constraints (civil engineering degree). The third group, “Others”, had no prior knowledge of either anatomy or mechanics. In Belgium, an experienced orthopaedic and trauma surgeon performed the same surgery three times to provide a reference to serve as the “surgical control”. He was experienced with the classical Hoffmann® external fixators^30–32^ but not the EZExFix. He therefore received the same pre-surgery theoretical information as the astronauts would receive before performing the surgery.

#### Experimentations

On day one of the mission, the three groups first attended a quick one-hour theoretical course on indications, anatomical landmarks and steps to attach the EZExFix followed by a practical demonstration.

**Figure 1:**
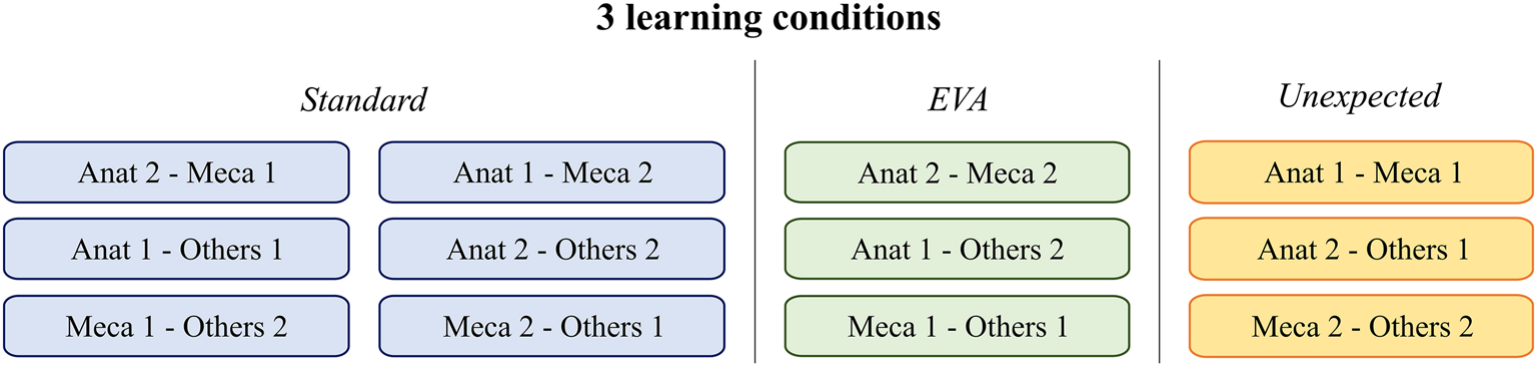
Organization and crossmatch of all operators depending on their skill group and the different learning conditions. Astronauts were divided into 3 groups according to their educational background. Anat: analogue astronauts skilled in human anatomy (studies in the medical or biomedical field but not in surgery), Meca: knowledge of mechanics, stability, forces movements and constraints (engineers), Others: no prior knowledge of either anatomy or mechanics. Learning conditions included standard conditions and stressful conditions (unexpected moment and during EVA: Extravehicular Activity).

Subsequently, each astronaut had to perform all eight tasks one after the other, sometimes as the operator placing the EZExFix on the broken leg (referred to as “Anat 1” or “Anat 2” depending on the person in the “Anat” group), sometimes as the assistant, helping to maintain the fracture reduction. Two surgeries were performed simultaneously by two astronauts during which they had to set up the EZExFix to repair an artificial broken leg, in the most efficient and quickest way, similar to a time trial. Twelve different combinations, or blocks, were therefore needed to crossmatch all operators (Fig. 1). Each astronaut performed the surgery four times as the operator and four times as the assistant leading to a total of 24 surgeries for all six astronauts. The different groups can also be compared in terms of skills to assess the need for prior basic skills in anatomy or mechanics. The Fig. 1 illustrates how the study design can integrate as well as the background knowledge (name of the group), the confrontation with every different operator and three different progressive learning conditions (different colours).

#### Learning conditions

Given a fracture occurring in space could induce stress in the crew, different conditions were applied to detect possible differences in performance. Carrying out each surgery as a competitive timed trial highlighted the importance of time and potentially induced a stress on the two operators competing. The stress level was also changed during the mission by scheduling the surgeries under three different conditions (Fig. 1). Standard conditions (blue) implied that all equipment was already prepared laid out on a table. All astronauts had to carry on at least one surgery under standard conditions before performing it under stressful conditions. Stressful conditions were obtained by executing the surgery either during an extravehicular activity (EVA) wearing space suits (green) or at an unexpected moment, such as during the night or at mealtimes, with nothing prepared (yellow). For the sake of homogeneity, the Fig. 1 allowed to organize the time trials so that each astronaut can perform the surgeries against each member of the other two groups and so that they can perform them twice under standard conditions and two times under stress conditions (EVA and unexpected moment).

#### Operations scheduling

In summary, all operational constraints included four surgeries for each astronaut, all possible combinations of operators, two standard conditions and one of both stressful conditions for each astronaut, with at least one trial in standard conditions before any stressful condition. All the trials have to be completed within the two weeks of the mission.

The latter encompassed eight scientific projects alongside the EZExFix project, posing the challenge of coordinating schedules to accommodate operational restrictions and shared constraints. This complex combinatorial issue was addressed using an AI system, Romie^33^. This system not only initially devised an optimal mission schedule but also continually adjusted and improved the scheduling of remaining simulation days based on real-time outcomes, all aimed at enhancing mission success probabilities.

### Fractured leg model

**Figure 2:**
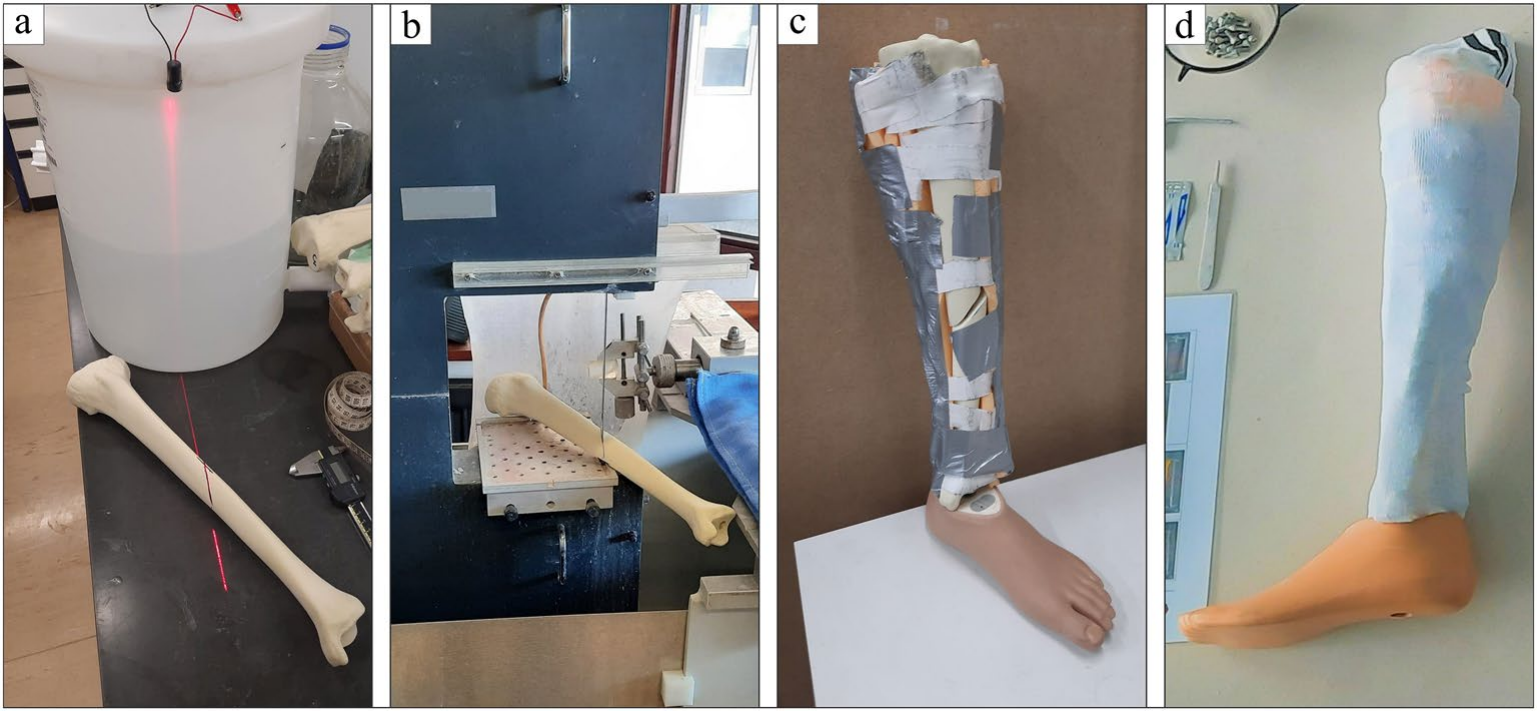
Creation of the fractured leg model. Indication of the fracture line with a laser (**a** – red line). Bone cutting by the diamond bandsaw following the fracture line (**b**). Soft tissues assembly and fixation around the fractured bone, mounted on a foot prosthesis (**c**). Final fractured leg model (**d**).

The bone model used to reproduce a fractured leg was that of a left tibia with cortical hard density and low cancellous bone structure, a pre-drilled intramedullary canal and a distal opening (LSH1385, Synbone SDN BHD, Malaysia). A simple oblique fracture line was created in the middle of the tibial diaphysis by using a laser and diamond bandsaw so as to always reproduce the same AO/OTA 42A2 closed fracture type following AO classification (Fig. 2). Soft tissues made from foam rubber sheet (22,320, Komprex®, Lohmann & Rauscher, Germany) were fixed around the bone to produce the shape of the leg and were covered by a sock to mimic the skin. The distal opening was used to attach a prosthetic foot in order to provide an indication of the axis in the event of further realignment surgeries. Each surgery required a new leg model.

### External fixator

**Figure 3:**
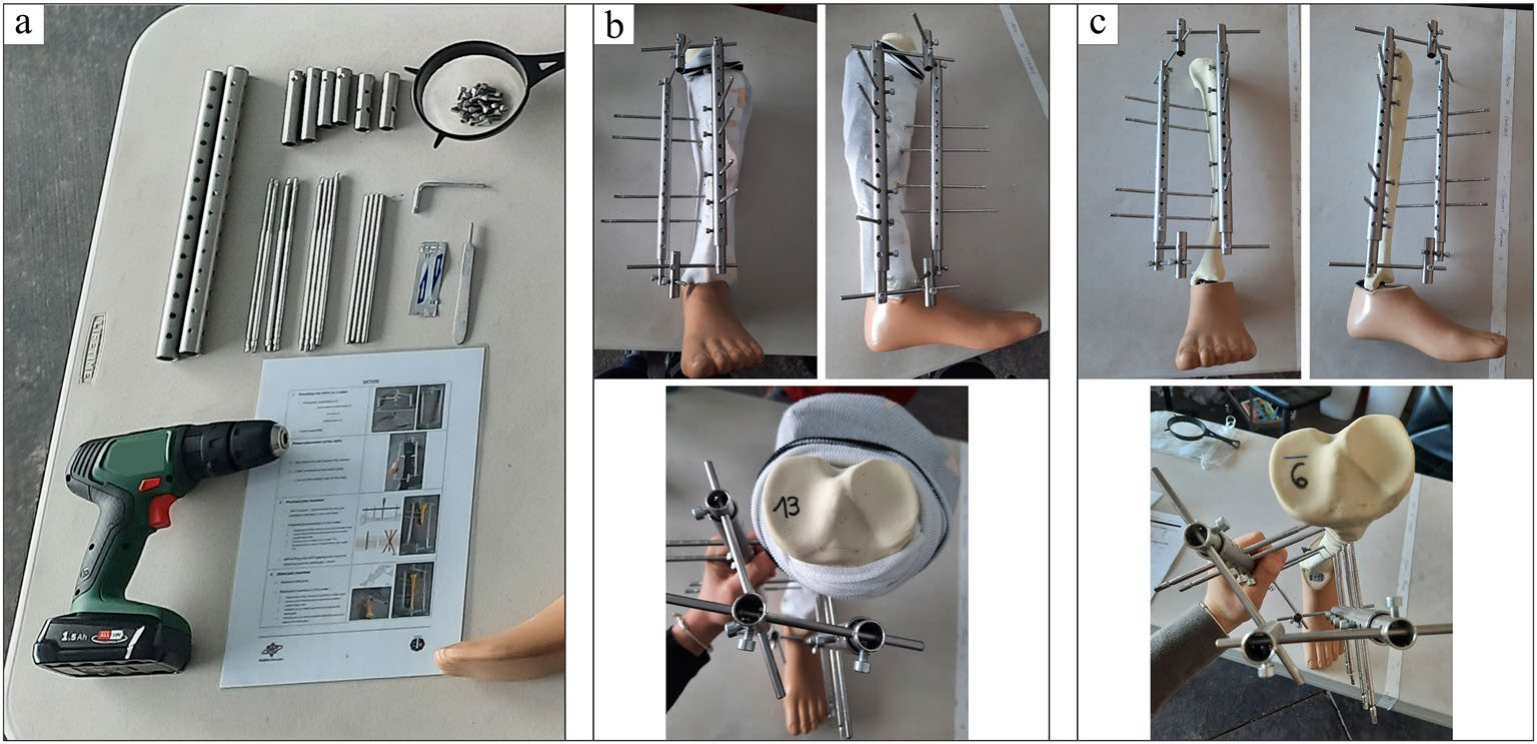
Material needed to build the EZExFix (**a**). Final construct mounted on a broken artificial leg on a frontal, sagittal and upper view (**b**). Broken artificial leg after removing soft tissues ready to measure analysis parameters on a frontal, sagittal and upper view (**c**).

The EZExFix concept considers both the mechanical stability needed to treat a long bone fracture and the ease of execution. A new unilateral biplanar EZExFix was developed in collaboration with orthopaedic surgeons from the Cliniques Universitaires Saint-Luc (Brussels) and engineers from ECAM (High Industrial Institute, Brussels). This new technology’s core focus was to be a low-cost, fast and easy-to-use fixator to extend the use of this treatment to hostile environments^28,34–36^. Figure 3 illustrates both the parts needed to build the EZExFix and the final assembly. This device can be used to treat all types of tibial shaft fractures from the simplest to more complex and is also suitable for treatment of significant soft tissue lesions. Mechanical properties were previously validated and are similar to the Hoffmann® 3 fixator (Stryker Trauma AG, Selzach, Switzerland)^29^.

A Practical Quick Guide containing the key steps needed to place the EZExFix was written to help astronauts carry out their tasks (Suppl. Figure 1). Two EZExFix were created to allow face to face surgeries. Soft tissues can then be removed from the bone while keeping in place the EZExFix, which can be disassembled after data harvesting and reused for subsequent surgeries.

### Analysis parameters

All parameters were examined during each experiment and for each operator. They were noted on a scorecard (Suppl. Figure 2) by a neutral examiner, someone not involved in performing surgeries. This neutral examiner remained always the same person for all tests to ensure comparative results. The scorecard facilitated parameters validation and the identification of potential mistakes or shortcomings. A video of each timed trial was simultaneously recorded for more accurate observation when required.

#### Safety

Patient safety is paramount and astronaut operators must not damage blood vessels, nerves, or tendons of their crewmates. Safe zones, described for the classical external fixation technique^37,38^ were assessed with the scorecard including five analysis criteria (Suppl. Figure 2). These safe zones are the tibial crest and the anteromedial side of the tibia, as well as the pins position with respect to joints and fracture lines. Range of movement has to be preserved; this was done by not directly inserting pins into the joint whilst also avoiding any areas that are required to move. For this reason, pins could only be inserted in the bone diaphysis. The depth of pins was also evaluated because an over-screwing by 5 mm can damage structures behind the second cortex, such as blood vessels or nerves. Each safe zone was registered as correct or not, and the sum of safe zones respected for each operation was calculated.

#### Procedure steps

To devise an efficient set up, steps had to be performed in a specific order (Suppl. Figure 1). Four main steps were determined, and sub-criteria for each step were established in order to determine the failure or success of each step. Since the EZExFix is limited to certain degrees of freedom, the triangulation and the frame had to be built in the first step. Then, the correct positioning of the EZExFix on the broken leg was essential in order to check the pins’ orientations, the respect of safe zones and the absence of compression point on the skin. The width of the incision in the skin (≤ 2cm), and the order in which pins were inserted were evaluated in the third step and noted on the scorecard by the neutral examiner. The total number of pins and the stability of fracture reduction were assessed in the fourth step. Skin compression and stability were both defined as main criteria directly influencing patient comorbidities and outcomes. These criteria were considered as a condition sine qua non to ensure a healthy evolution of the broken leg. Each step was defined as a success or a failure, and the sum of step outcomes was calculated for each surgery.

#### Time

The total time taken by each operator to complete the task was taken by the neutral examiner, as well as intermediate times for each step, expressed in minutes. The surgeries started upon instruction from the neutral examiner.

### Statistical analysis

Quantitative variables were analysed in terms of central tendency (mean and median) and dispersion by the range (minimum–maximum) due to the small sample size. The normality of distributions was verified using QQ plots to determine whether to proceed with parametric or nonparametric tests. Comparisons between educational background and learning conditions were evaluated by one-way ANOVA for a quantitative variable (time). The homogeneity of variances was examined by Levene’s test. The same comparisons were evaluated by a Poisson regression for discrete variables (safe zones and steps) in order to detect a main effect and/or an interaction between both factors. The absence of overdispersion was verified by a Chi-squared test. In order to compare the different durations of each step within the astronaut group, a one-way ANOVA for repeated measures was performed. The sphericity of the variance–covariance matrix was evaluated by Mauchly’s test. The normality of residuals was verified by QQ plots for both ANOVA tests. All generalized linear models integrated multiple comparisons if justified, adjusted with a Bonferroni correction. The time comparison of each step between astronauts and the surgical control was performed with non-parametric two-tailed Mann–Whitney tests because of the small number of samples. The level of significance was always set to 0.05 in order to reject the null hypothesis. All statistics were performed using SPSS software (V.27, IBM SPSS, Inc., Chicago, IL, USA).

## Results

### Safety: safe zone respect

Across the 5 defined safe zones, on average four were respected systematically by each operator (median 4.0; range 2–5) (Table 1).

**Table 1:**
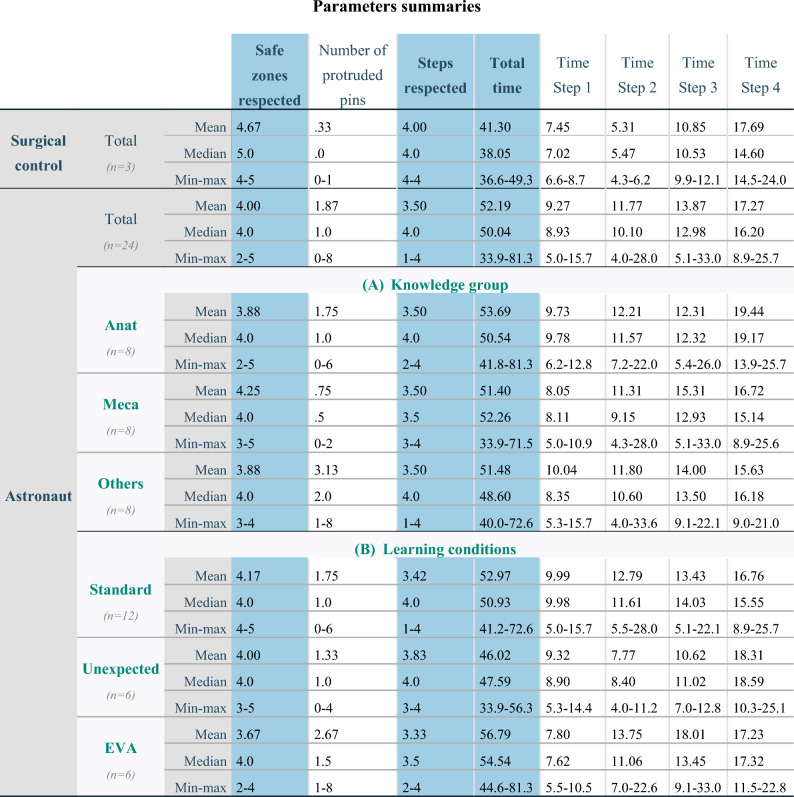
Descriptive analysis of three different parameters: safe zones respected, steps respected and total surgical time (blue background). Quantitative variables were analysed in terms of central tendency (mean and median) and dispersion by the range (Min–max). Some meaningful sub-criteria depending on those main three parameters are also mentioned and described (white background). Descriptive data from astronauts are subdivided according to their knowledge group (A) and their learning conditions (B) (both in bold green). Times are expressed in minutes. n: sample size. EVA: extravehicular activity.

**Figure 4:**
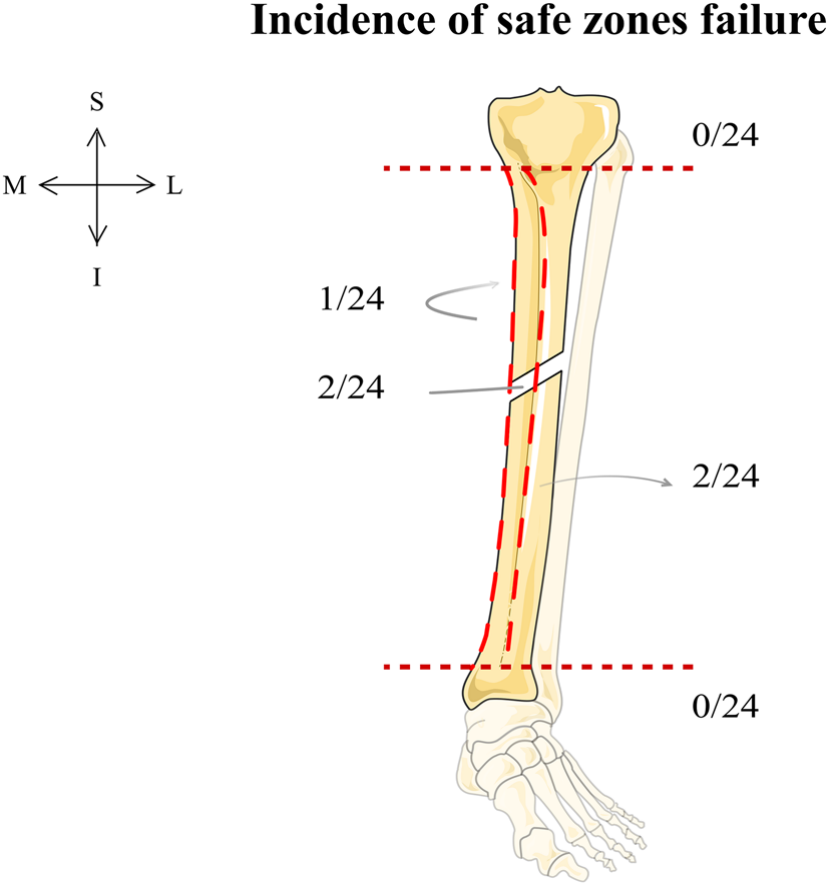
Incidence of safe zones failure among the 24 operations. Horizontal lines indicate the metaphyseal limit and vertical lines demarcate the safe surface between the tibial crest and the medial border of the tibia surrounding the anteromedial side of the tibia.

Pins were never inserted in the articular joints (0/24) and almost never in the fracture line (2/24), outside of the tibial crest (2/24) or outside the anteromedial side of the tibia (1/24) (Fig. 4).

Pin protrusion was the criterion failing most frequently (19/24 regardless of progress because at least 1 pin protruded more than 5 mm during 5/6 operations on the first as well as on the fourth trial). The mean number of protruding pins was 1.87 (median 1.0; range 0–8). From the three trials, the surgical control made only one pin protrusion on the first trial whilst respecting all other criteria. The different educational backgrounds of the astronauts, as well as the stress conditions, did not affect compliance with safe zones (Table 2-a).

**Table 2:**
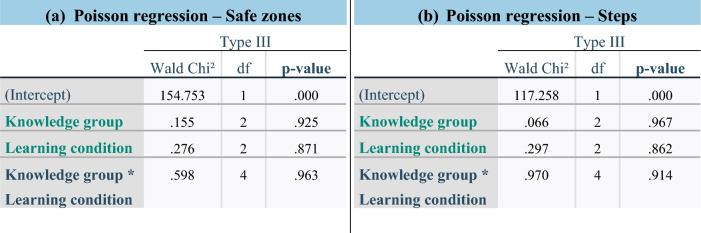
Poisson regressions testing model effects. Different dependent variables included respecting safe zones (a) and steps (b). df: degree of freedom.

### Procedure steps

**Figure 5:**
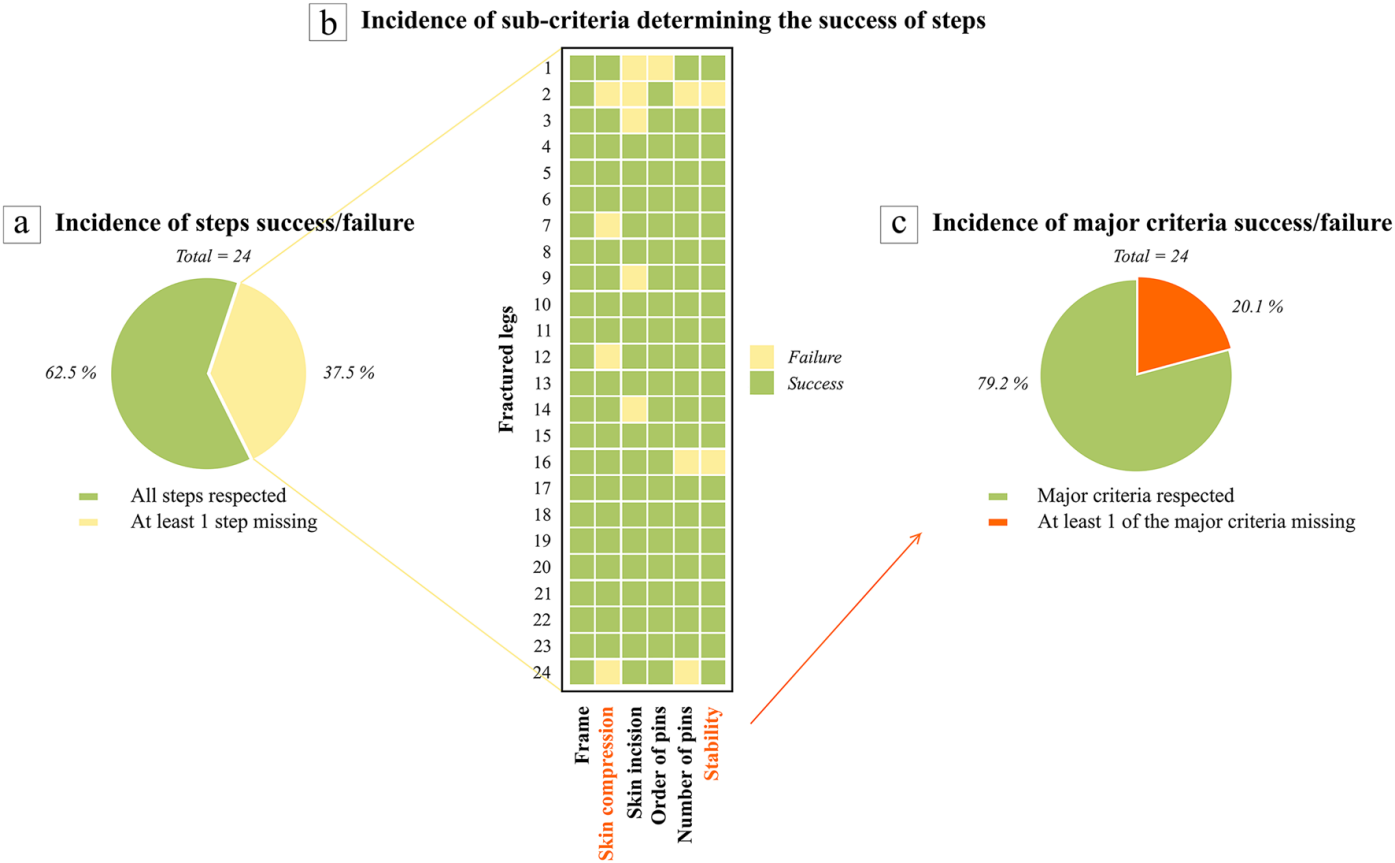
Incidence of steps success/failure among the 24 astronauts’ operations taking all sub-criteria into account (**a**) and repartition of success/failure following each sub-criterion (**b**). Both main sub-criteria to ensure a healthy evolution are font coloured in orange. Incidence of steps failure among the 24 astronauts’ operations when only main sub-criteria are considered (**c**).

From the four scorecard steps, the astronauts satisfied on average 3.5 (median 4.0; range 1–4) (Table 1), whilst the surgical control always satisfied all four steps. Among all operations, 62.5% of astronauts performed all steps correctly (Fig. 5-a). The frame assembly was always made properly during the first step (24/24) (Fig. 5-b). Pins were placed correctly, such that the skin was not compressed, in 20/24 operations. The step called “proximal pins” in the Practical Quick Guide was successful in 19/24 operations but failed five times due to too wide a skin incision (> 2 cm). Respecting the order of pin insertion failed only once, but on three occasions fewer than eight pins were actually inserted. The “distal pins” criterion was not met three times (3/24), from which the pins count failed twice to ensure fracture stability. When only main criteria are considered, the success rate reached 79.2% (Fig. 5-c).

The different educational backgrounds amongst the astronauts did not affect their success at performing the steps because they all achieved a mean of 3.5 correctly performed steps (Table 1). The increasing stress did not alter the sequence for astronauts (Table 2-b).

### Time

**Figure 6:**
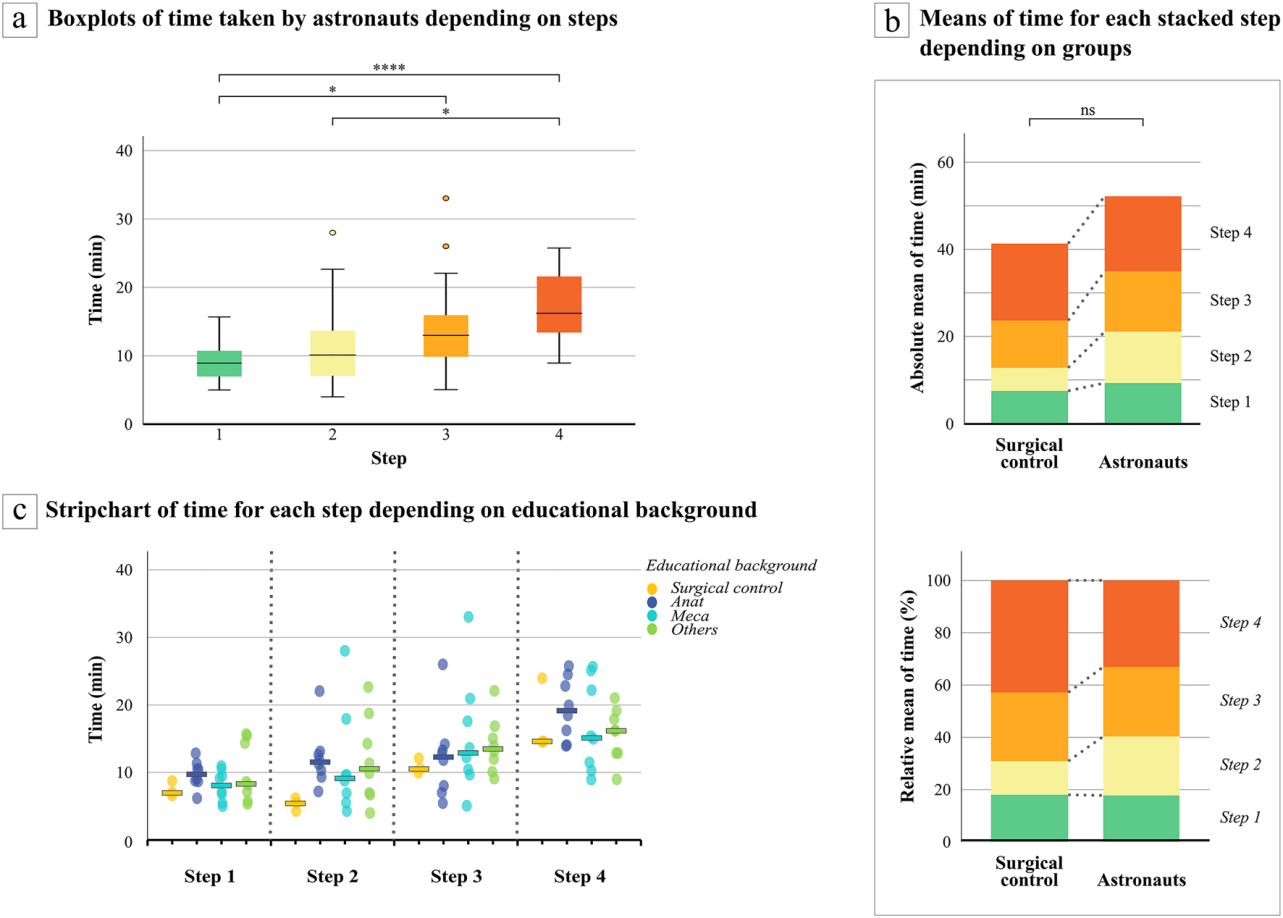
Boxplots of time taken by astronauts depending on steps (**a**). Center line: median, box limits: upper and lower quartiles, whiskers: 1.5 × interquartile range, °: outliers, Min: minutes, *: p < 0.05, ****: p < 0.0001 from one-way ANOVA test. Graphs of absolute and relative means of time for each stacked step depending on groups (surgical control or astronauts) (**b**). Dotted grey lines (…): lines connecting steps between both groups suggesting a difference when lines are not parallel between lower and upper border of a step or a similarity if both lines are parallel. Min: minutes, ns: non-significant. Strip chart of time required for each step depending on educational background (**c**). The different educational background between astronauts did not affect neither the total time (p = 0.904) nor the time at each step (p = 0.396, p = 0.961, p = 0.669, p = 0.340 from step 1 to 4 respectively) following the one-way ANOVA test. Min: minutes. Bars: medians.

Descriptive analyses (Table 1) showed that an astronaut took on average 52.19 min (median 50.04; range 33.9–81.3) to perform the complete task while the surgical control took only 41.30 min (median 38.05; range 36.6–49.3). The time analyses between different steps within the astronaut population highlighted some statistical differences. Figure 6-a shows a progressive increase in the median time with increasing step number. The one-way ANOVA (Table 3) demonstrated a significant difference between step times, and pairwise comparisons highlighted that step four was significantly longer than step 1 (p = 0.000008) and step two (p = 0.018), and also that step three was longer than step one (p = 0.021).

**Table 3:**
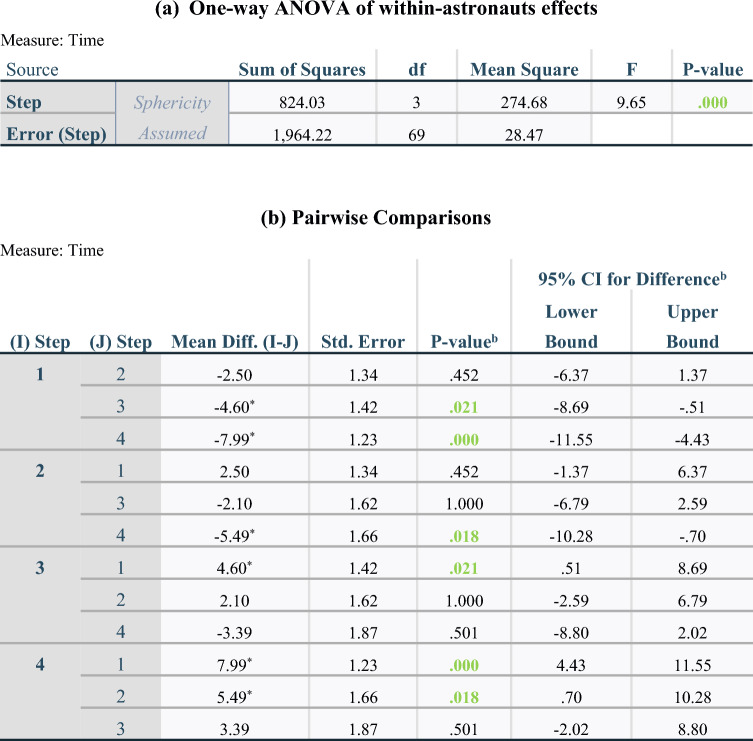
One-way ANOVA test for repeated measures determining a difference between steps within astronauts’ group (a). Mauchly’s test assumed the sphericity of the variance–covariance matrix (p = 0.215). Pairwise comparisons to highlight which steps are different from others within the astronaut population (b). df: degree of freedom, F: value on the F distribution.

Based on estimated marginal means.

*The mean difference is significant at the .05 level.

^b^Adjustment for multiple comparisons: Bonferroni.

Figure 6-b, a stacked bar graph comparing both groups (astronauts and surgical control), illustrated that the most important difference in time was in the second step. Even when the surgical time is visualized by its absolute or relative values, the second step was always longer for astronauts than for the surgical control (non-parallel dotted lines). However, this was not statistically confirmed by the Mann–Whitney test (p = 0.063) (Table 4). Regarding only relative averages of time, the fourth step was the longest for the surgical control.

**Table 4:**
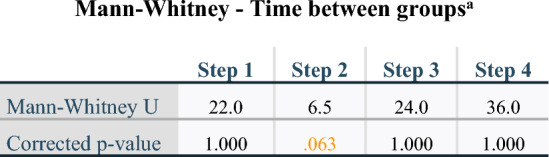
Mann–Whitney tests comparing central tendency of time between groups (surgical control or astronauts) for each step separately after a Bonferroni correction.

^a^Grouping variable: Astronaut or surgical control.

The different educational backgrounds of the astronauts did not affect the total time (p = 0.904) nor the time for each step (p = 0.396, p = 0.961, p = 0.669, p = 0.340 from step 1 to 4 respectively) (Fig. 6-c). The same conclusion was reached for the stress condition (p = 0.237 for total time and p = 0.376, p = 0.175, p = 0.133 and p = 0.851 from step 1 to 4 respectively).

## Discussion

Despite astronaut selection being very competitive, including healthy people meeting NASA’s strict Space Flight Human System Standards for Crew Health (NASA-STD-3001)^8,10,39^, spaceflight accelerate the body’s senescence. Prevention strategies to limit the BMD loss are unavoidable and have been explored extensively in the literature, but are also fallible^7^. Nonetheless, detail about fracture management in spatial missions is lacking in the literature^1^. This study did not address osteoporosis prevention but focused on a solution to treat a consequence of LDEM by learning quickly how to use an EZExFix to fix long bone fractures.

### Safe zones

Initially, astronauts could consult the Practical Quick Guide (Suppl. Figure 1) to help them. However, from the third trial, it was no longer consulted. Safe zones were almost always respected, ensuring a safe surgery for patients. The only criterion that could generate a problem was the protrusion of pins. Pins were self-drilling and self-tapping and were thus inserted without pre-drilling, meaning astronauts were not able to feel the right depth. Actually, pre-drilling allows surgeons to feel the passage through the second bone cortex and offers the possibility of measuring the depth to which the pin should be driven, without recourse to radioscopy. This step was not integrated in this procedure in order to minimize the time and the number of surgical instruments needed but could be implemented easily. Another option could be the use of a pinless fixator^40,41^. Unicortical external fixators have also been developed avoiding cortex protrusions and limiting deep infection complications^42^.

### Workflow

Concerning the steps and major sub-criteria required to achieve fracture healing, astronauts reached a success rate of nearly 80%, meaning that the EZExFix is a good device, easy to use and to learn for the purpose of repairing long bone fractures. Nevertheless, improvements in both major and minor sub-criteria are still possible. Minor criteria could be enhanced by taking precautions when drilling. In practice, the artificial leg skin, represented by a sock, is prone to wrap around the pin when drilling leading to too wide a skin incision. However, in a real operating theatre, the same mechanism can occur with soft tissues between skin and bone. This issue could be addressed simply by using a protective sleeve to separate the pin’s thread from soft tissues.

### Manipulation time

Analogue astronauts took longer (mean 52.19 min, median 50.04; range 33.9–81.3) than the surgical control to place an EZExFix but followed the mean operating time in the literature. Different authors described a range of mean times to place an external fixator on a femur or tibia of between 35 and 74.6 minutes^43–45^. However, when analysing separate steps, astronauts took significantly more time for steps three and four than first two steps. The step that showed the most difference in time between the astronauts and the surgical control was the second step. Astronauts spent more time performing the second step to place correctly the EZExFix and to avoid skin compression, which is a main sub-criterion influencing good evolution. The longest step for both astronauts and surgical control was the fourth step, including fracture reduction and stabilization which is the second main sub-criterion^46^. Thus, astronauts automatically became aware of the paramount relevance of these two main criteria to rescue their patients, whereas surgeons only need to focus on the fourth step, probably because the second one is more easily acquired by their own experience. One limitation in interpreting these results is that the variance of the surgical group was reduced to its within operator variance, whereas the variance of the astronaut group was made up of within and between operator variances. For this reason, comparisons between the astronauts and the surgical group give an idea of what can be expected or what the astronauts should achieve but should be interpreted with caution.

### Training time

The literature already made the link between the selection and training of surgeons and astronauts^47^, both needing a high level of prerequisites in terms of KSAOs. The need for a medical expertise seems to increase with the mission duration and the size of the crew^20^. To ensure the safety and maintenance of the crew without a physician on board, selected crew-members require medical training to manage pathological situations^20^, such as bone fractures or emergencies. The time and the ease of learning a useful technique is essential to maximize the efficacy and the cost-effectiveness of an astronaut education. This study showed that the training accomplished within two weeks while doing other scientific activities was enough to make an astronaut autonomous in tibial shaft oblique fracture treatment with the EZExFix device, despite an increase in stress conditions. The educational background did not influence outcomes, suggesting that only the quick theoretical course at the beginning was sufficient to understand and adequately perform the task irrespective of the astronaut’s basic knowledge. This observation means that the KSAOs initial requirements could be revised or adapted for astronaut’s selection because all astronauts could reach some skills and abilities in a very short period and without previous extensive knowledge.

### Limitations and further work

One limit of this study was the small number of subjects. Since the space analogue habitat is not compatible with many subjects, one way to address this would be to repeat the experiments in subsequent missions to supports these results. Other types of fractures could also be considered in forthcoming missions, such as wrist fractures.

Sterility, not evaluated in this study, could be considered in future studies. Despite the ease of the EZExFix procedure, sterility is a recommended parameter because it remains an aseptic surgical procedure. However, the EFORT (European federation of national associations of orthopaedics and traumatology) open reviews allowed the use of external fixator in the emergency department for life-threatening patients with pelvic, tibial, femoral or humeral instability^37^. Subsequent studies should collectively incorporate additional parameters (e.g., diagnosis, sterility as well as the set up of a suitable surgical environment), as the need to react to an emergent situation might impact the pace at which the participants perform the required tasks.

One disadvantage of the EZExFix is the difficulty to maintain a pressurized suit. Further research is required to evaluate the possibility of reducing the size of the EZExFix until it can fit inside the suit without loosening its mechanical properties, or of adapting the suit with a larger leg or to compartmentalize pressurized zones. Currently, this device is not suitable in EVA and repatriation into the pressurized habitat would be mandatory.

The EZExFix is a surgical technique that typically encourages secondary healing with callous formation. Nonetheless, since the fracture callus creation can be smaller and weaker in low gravity conditions^1,23^, astronauts should consider realigning the fracture and applying compression before securing the distal part of the construct in order to maximize the potential for direct bone healing. As the comprehension of fracture repair mechanisms in low gravity environment remains uncertain^9,22,23^, factors influencing direct or indirect bone healing required further investigation.

A major concern is taking care of soft tissue (especially in open fractures) and wound healing, which could be impaired in space conditions by increasing cell apoptosis, inflammation and decreasing matrix formation^48,49^. While allowing preservation of fracture hematoma, external fixation is also less invasive than other surgical fixation methods thereby decreasing bleeding, infection risk and comorbidities. It is also possible that a non-surgeon astronaut could use it without extensive surgical training^1,50^. This technique could also be performed under local or locoregional anaesthesia, which are preferable in space due to their safety, fast recovery, ease of use, antagonist availability and smaller equipment needed while avoiding endotracheal intubation and hazardous manipulation of volatile gaseous anesthetics^9,50^. The onboard supplies have to be scheduled strategically in advance in order to minimize the mass, the size and the amount of equipment and maximize its function and efficiency^2,21^. The storage space required for an EZExFix would not exceed 35 × 20 × 10 cm and could be stored readily. A correctly executed EZExFix procedure could certainly allow weight-bearing on Mars because mechanical tests on Earth were satisfactory^29^. Weight-bearing is of primary importance for the mission success, autonomy and promotes faster consolidation^1^ than is possible with a cast. External fixation is the only orthopaedic therapy that offers all these benefits in one device.

## Conclusion

While LDEM may lead to a decrease in BMD and potentially increase the risk of fractures, the ability to autonomously manage long bone fractures is crucial for the success of such missions. In cases where a lower extremity fracture of the tibia is suspected, the accurate diagnosis should be made. Immediate on-site imaging techniques like ultrasounds can help confirm the diagnosis and determine the need for an EZExFix. Ideally, the injured astronaut should be transported to a secure location, such as the back of the pressurized rover or the main base, where local anaesthesia can be administered, and the injury site sterilized to the best possible extent before the fixation procedure is performed. Subsequently, astronauts would be able to autonomously fix a tibial shaft fracture with an EZExFix. This study is encouraging for space exploration because this device could be considered as safe, easy, quick and efficient to treat astronaut fractures, in an autonomous way, without ground assistance, without the deployment of large equipment, without the need for a medical/surgical or mechanical background, and with only a few days of training. Similar techniques could be used in LDEM to increase astronauts’ autonomy in traumatic injury treatment and lower skill competency requirements used in crew selection.

### Supplementary Information

Supplementary Information.

## Data Availability

All data analysed during the study are included in this published article. The complete original datasets generated during the current study are available from the corresponding author on reasonable request.
