# Supplementary material for: Adequacy of in-mission training to treat tibial shaft fractures in mars analogue testing
Source: Sci Rep. 2023 Oct 23;13:18072. doi: 10.1038/s41598-023-43878-1 (PMC10593937; doi:10.1038/s41598-023-43878-1)

**Adequacy of In-Mission Training to Treat Tibial Shaft Fractures in Mars Analog Testing**

SUPPLEMENTARY INFORMATION

***Supplementary figure 1.*** *Practical Quick Guide to expose material, safe zones and method in order to use the external fixator. Safe zones adapted from Checketts, R.G. and C.F. Young, (iii) External fixation of diaphyseal fractures of the tibia. Current Orthopedics, 2003. 17(3): p. 176-189.*


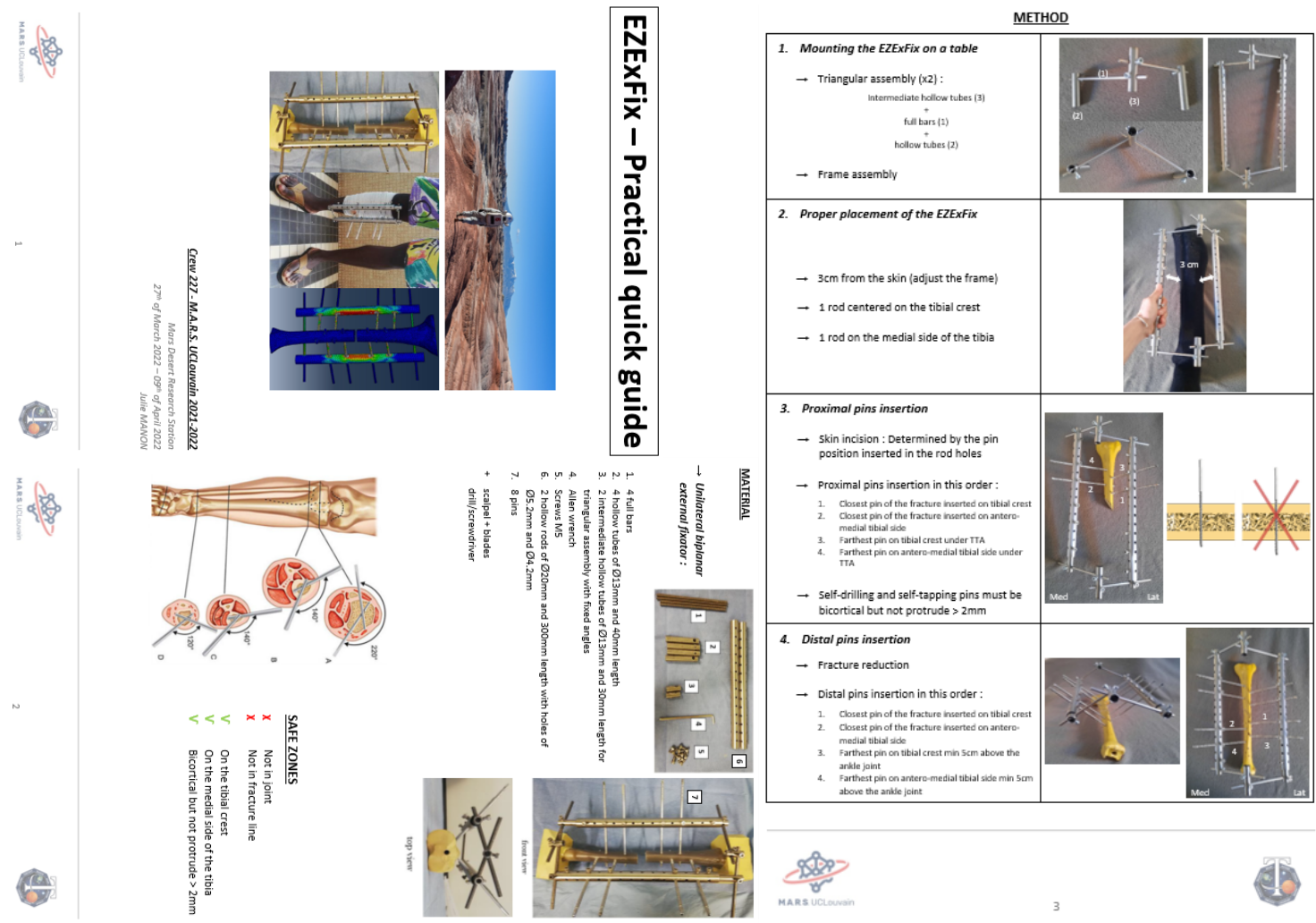


***Supplementary figure 2.*** *Scorecard used to harvest data from all surgeries. This allows to underline safe zones and different respected steps as well as the time associated with each step.*


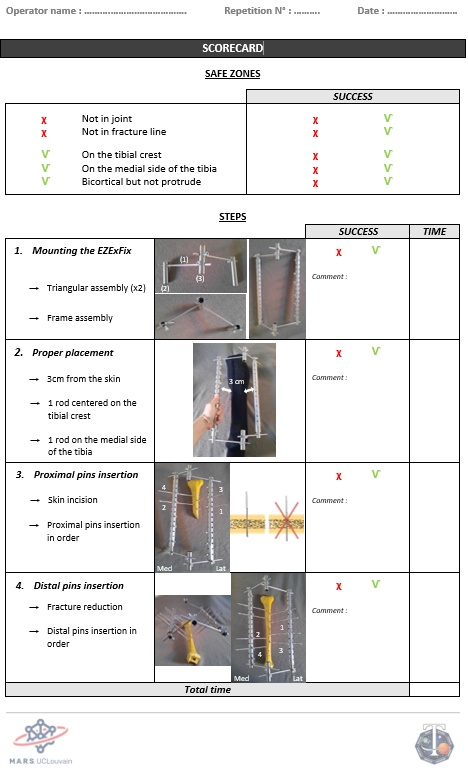

Supplement: Supplementary file 1 — Supplementary Information. [file 41598_2023_43878_MOESM1_ESM.docx]
